# Influence on the fermentation quality, microbial diversity, and metabolomics in the ensiling of sunflower stalks and alfalfa

**DOI:** 10.3389/fpls.2024.1333207

**Published:** 2024-01-26

**Authors:** Heng Jiang, Si-Yi Wang, Hao-Ran Wang, Yuan-Yuan Jing, Hui Qu, Le Sun, Jiao Wang, Bin Liu, Feng-Qin Gao

**Affiliations:** ^1^ Institute of Grassland Research Chinese, Academy of Agricultural Science, Hohhot, China; ^2^ College of Grassland Science, Qingdao Agricultural University, Qingdao, China; ^3^ Animal Husbandry Institute of Inner Mongolia Academy of Agricultural & Animal Husbandry Sciences, Hohhot, China

**Keywords:** sunflower straw, alfalfa, silage, fermentation, bacterial community, metabolites

## Abstract

With the rapid development of the livestock industry, finding new sources of feed has become a critical issue that needs to be addressed urgently. China is one of the top five sunflower producers in the world and generates a massive amount of sunflower stalks annually, yet this resource has not been effectively utilized. Therefore, in order to tap into the potential of sunflower stalks for animal feed, it is essential to explore and develop efficient methods for their utilization.In this study, various proportions of alfalfa and sunflower straw were co-ensiled with the following mixing ratios: 0:10, 2:8, 4:6, 5:5, 6:4, and 8:2, denoted as A0S10, A2S8, A4S6, A5S5, A6S4, and A8S2, respectively. The nutrient composition, fermentation quality, microbial quantity, microbial diversity, and broad-spectrum metabolomics on the 60th day were assessed. The results showed that the treatment groups with more sunflower straw added (A2S8, A4S6) could start fermentation earlier. On the first day of fermentation, *Weissella* spp.dominated overwhelmingly in these two groups. At the same time, in the early stage of fermentation, the pH in these two groups dropped rapidly, which could effectively reduce the loss of nutrients in the early stage of fermentation.In the later fermentation period, a declining trend in acetic acid levels was observed in A0S10, A2S8, and A4S6, while no butyric acid production was detected in A0S10 and A2S8 throughout the process. In A4S6, butyric acid production was observed only after 30 days of fermentation. From the perspective of metabolites, compared with sunflower ensiling alone, many bioactive substances such as flavonoids, alkaloids, and terpenes are upregulated in mixed ensiling.

## Introduction

Sunflower (*Helianthus annuus L.*) is widely distributed throughout the world as one of the most important oilseed crops for its short growth cycle, drought tolerance, barrenness and salinity (Eltz et al., 2010; Tan, 2015; Ortiz-Hernandez et al., 2020). However, with a large number of seeds provided, sunflower produces a large amount of sunflower straw. According to statistics, the global planted area of sunflowers were more than 26.5 million hectares in 2017, generating about 80-186 million tons of remaining straw (Mehdikhani et al., 2019). In China, sunflower is a widely planted oilseed crop, and its straw production is about 12 billion tons (Rocha et al., 2004). However, due to the inefficient management practices of using sunflower straw, a large amount of this biomass is often incinerated in vast agricultural areas of China, which not only leads to environmental pollution, but also causes a great waste of biomass resources (Yue et al., 2018).

Many studies have shown that as a result of sunflower’s high energy value and crude protein, it has great feeding potential, and where is not suitable for planting corn silage, sunflower silage can be used as a supplement of maize silage (Tomich et al., 2003; Neumann et al., 2013). Some scholars have found that the feeding value of sunflower silage can be up to 80% of maize silage (Thomas et al., 1982). Compared to sorghum silage, sunflower silage is higher among ether extract, mineral content and crude protein (Rocha et al., 2004). However, sunflower silage has a higher content in acid detergent fiber and lignin than alfalfa and maize silage, in this condition it can not completely replace normal dietary feeds (Pereira et al., 2007). Gholami-Yangije et al. (2019) thought that there was no effect on milk production and composition of dairy goats when sunflower silage replaced a certain percentage of diet. Therefore, in order to reduce the negative effects of sunflower silage, there may be a feasible solution in using sunflower straw mixed with high quality pasture in specific proportions for silage. Alfalfa is an essential forage used for its high nutritional value, good palatability and digestibility, but due to the small number of endophytes and lacking substrate of fermentation alfalfa is define as a hard-ensile crop. There have been a lot of studies on using alfalfa and waste resources of agriculture for mixed silage to produce high-quality feeds recently, therefore it seems to be feasible that using alfalfa and the straw of sunflower for mixed silage (Wang et al., 2021b; Chen et al., 2023).

Due to different buffer energy values、microorganisms carried by different raw materials and fermentation substrate contents among different raw materials, the microbial community succession and microbial interactions will be different. The effect be on fermentation quality and nutritional value of microorganisms in silage would be significant, so it is necessary to clarity the process of microbial community succession during mixed silage fermentation. At the same time, different raw materials as well as different microbial diversity induce different metabolites in each mixed silage. Metabolites not only affect flavor and fermentation quality, but also affect the palatability of livestocks and nutrition (Du et al., 2022). Therefore, in order to determine the fermentation end-products, metabolomics is considered to be a powerful tool to study metabolites during silage fermentation (Su et al., 2023). However, the studies on sunflower straw silage have been limited to its fermentation quality and nutritional value so far, while microbial community succession, microbial interactions and their metabolites during fermentation are not clear at the moment. In view of this, it is necessary to study the effects of mixed silage between sunflower straw and alfalfa, especially the differences in microbial composition and metabolomics between individual silage and mixed silage.

In this study, a mix composed by different proportions of sunflower straw and alfalfa as silage has been proposed to evaluate the nutritional quality, fermentation patterns, microbial diversity and metabolomics asserting the optimal mixing ratio as silage, providing technical support for the application of sunflower straw in silage without affecting animal performances. At the same time, more in-depth researches will be conducted through microbiome and metabolomics, and it will provide useful information for a better understanding of biochemical process in silage.

## Materials and methods

### Preparations before silage

Sunflower and alfalfa were planted in Tumd East Banner, Hohhot, Inner Mongolia Autonomous Region, National Modern Agricultural Demonstration Park in China’s Inner Mongolia, where the longitude is 111.388458 E, the latitude is 40.74075 N, the mean annual temperature is 6.3°C, and the mean annual precipitation is 400 mm. On October 2, 2022, the fourth cutting of alfalfa at the early bloom stage was harvested, along with the sunflower stalks that remained after the collection of the sunflower heads. Using CLAAS self-propelled silage harvester (JAGUAR 880, Germany) to harvest and pulverize the two raw materials to 2.5-3.5 cm, and then dry them 24-hrs naturally, leading to its moisture content to be reduced to 55-65%. Alfalfa and sunflower straw were mixed at weight ratios of 0:10 (A0S10), 2:8 (A2S8), 4:6 (A4S6), 5:5 (A5S5), 6:4 (A6S4), and 8:2 (A8S2) respectively. After thorough mixing, each treatment group was evenly sprayed with 0.005g/kg of ZhuangLeMei silage fermentation agent. (Sichuan Gaofuji Biological Technology Effective Co., Ltd., China.). (*Lactobacillus plantarum*≧1.3×10^10^CFU/g, *Lactobacillus brucei*≧7×10^9^CFU/g). Once mixed, an amount of 500g of raw material has been taken and put into a polyethylene bag (250 mm x 350 mm) to be vacuum-sealed and stored in a room at a monitored temperature ranged between 25-28°C. Each treatment made three replicates. The bags were opened to test the nutrient composition and fermentation quality at the 1st, 3rd, 5th, 7th, 15th, 30th, and 60th day of fermentation separately, and then measured the microbial population as well as the microbial diversity at the 1st, 5th, and 60th day of fermentation, respectively. Meanwhile, according to the data of fermentation indexes measured in the previous period, we chose to determine the broad-spectrum metabolomics of A0S10, A5S5 and A6S4 in the 60th day.

### Experimental index and measurements

#### Measurements of silage fermentation quality

After the silage samples were opened, 20 grams of silage was put into a beaker, 180 ml of deionized water was added and this was sealed with sealing film. This was placed in the refrigerator at 4°C for 24hrs and then it was taken out, this was then filtered through gauze which is four layers, and then through qualitative paper to get the extract. pH was obtained from extract with a portable pH meter (Laqua Twin, U.S.A). Ammoniacal nitrogen (NH_3_-N) content was determined by a phenol-sodium hypochlorite colorimetric method (Broderick and Kang, 1980). The contents of lactic acid (LA), acetic acid (AA), propionic acid (PA), and butyric acid (BA) were determinated by high-performance liquid chromatography (Waters 2695; USA; flow rate 1ml/min; temperature 30°C) according to Wang et al.’s (Wang et al., 2020) method.

#### Determination of microbial quantity

The determination of microbiological counts has been carried out weighting an aliquot of 10g from each fresh silage sample, that was punt into conical bottles containing 90ml of sterile water, sealed with a plastic film, put into a 180 r/min shaker for 30 mins to disperse the microbial cells, and then diluted into 10^-1^ – 10^-4^ dilutions after stewing for 10-40s. Lactic acid bacteria (LAB) were cultured in anaerobic conditions at 37°C for 48hrs with a MRS medium plate counting method, and taken 1ml the dilution solution with a concentration ratio of 1:10, added with 9ml of sterile water, and mixed thoroughly to make the dilution’s concentration ratio 1:100. Dilutions were made sequentially in a 10-fold gradient as described above. This took three compartments of petri dishes, which contained sterile MRS, Eosin-methylene Blue Medium and Rose Bengal Agar and marked the gradient of dilution; then sucked 20 μL from the corresponding dilution tubes with a micropipette, dropped it onto the corresponding fan-shaped area on the surface of the culture media, and then spread the bacterial solution evenly on the media with a spreading stick. The applied culture medium should be left to stand for 20-30 min and then inverted. All the media were placed in a 37°C constant temperature incubator for 48h and then counted.

#### Determination of silage fermentation quality

The remaining silage was dried to constant weight at 65°C and the dry matter (DM) content was determined, after which the samples were ground and passed through a 1 mm mesh sieve for chemical composition analysis. Water soluble carbohydrates (WSC) and crude protein (CP) contents were determined according to the Association of Official Analytical Chemists (AOAC) method (Hasan, 2015). Neutral detergent fiber (NDF) and acid detergent fiber (ADF) contents were determined by the method described by Van Soest et al. (1991). The evaluation of the EE content and Ash were performed using an XT15 extractor (Ankom), employing petroleum ether, as per the AOCS method (Firestone, 2009).

### Microbial diversity analysis

A 10 g sample was removed from each silage bag and 40 mL of sterile saline (0.9% NaCl) was added and mixed thoroughly by vortexing. The filtrate was centrifuged at 10,000 r/min for 10 min and the supernatant was discarded. The remaining precipitate was then suspended in 3mL of sterile brine. Microbial community total DNA extraction was performed according to the instructions of the E.Z.N.A.^®^ soil DNA kit (Omega Bio-tek, Norcross, GA, U.S.), and PCR of the V3-V4 variable region of the 16S rRNA gene was performed using 799F_1193R (5′-ACGTCATCCCCACCTTCC-3′). amplification, DNA extraction and PCR amplification were performed according to the method of Ni et al. (2017). Sequencing was performed using Illumina’s Miseq PE300/NovaSeq PE250 platform (Shanghai Meiji Biomedical Technology Co., Ltd.). PCR products from the same samples were mixed and recovered on a 2% agarose gel, purified using the AxyPrep DNA Gel Extraction Kit (Axygen Biosciences, Union City, CA, USA), detected by electrophoresis on a 2% agarose gel, and quantified by quantitative assay using the Quantus™ Fluorometer (Promega, USA). Fluorometer (Promega, USA) for quantification of the recovered products. Library construction was performed using NEXTflex™ Rapid DNA-Seq Kit (Bioo Scientific, USA).

The raw sequenced sequences were quality controlled using fastp (Chen et al., 2018) (https://github.com/OpenGene/fastp, version 0.20.0) software, and FLASH (Magoč and Salzberg, 2011) (http://www.cbcb.umd.edu/software/flash, version 1.2.7) software for splicing: using UPARSE (Edgar, 2013) software (http://drive5.com/uparse/, version 7.1), sequences were OTU clustered and chimeras were removed based on 97% (Stackebrandt and Goebel, 1994) similarity. Species classification was annotated for each sequence using RDP classifier (http://rdp.cme.msu.edu/, version 2.2), compared to the Silva 16S rRNA database (version 138), and the comparison threshold was set at 70%. All the sequences in the current study were deposited to the sequence read archive 156 (SRA) of the NCBI database under the accession number PRJNA1064671.

### Metabolomics analysis

The samples were placed in a lyophilizer (Scientz-100F) for vacuum freeze-drying; milled (30 Hz, 1.5 min) to powder form using a milling machine (MM400, Retsch); and then analyzed by extraction (-20°C pre-cooled 70% methanol aqueous internal standardized extracts), centrifugation (12,000 rpm, 3 min), and used for UPLC-MS/MS analysis.

The data acquisition instrumentation consists primarily of Ultra Performance Liquid Chromatography (UPLC) (ExionLC™ AD, https://sciex.com.cn/) and Tandem mass spectrometry (MS/MS).UHPLC separation was performed using an Agilent SB-C18 column (1.8 µm, 2.1 mm × 100 mm). Mobile phases: phase A was ultrapure water (with 0.1% formic acid added) and phase B was acetonitrile (with 0.1% formic acid added). The elution gradient was as follows: the B-phase ratio was 5% at 0.00 min, the B-phase ratio increased linearly to 95% within 9.00 min and was maintained at 95% for 1 min, and the B-phase ratio was decreased to 5% from 10.00 to 11.10 min and equilibrated at 5% for 14 min; the flow rate was 0.35 mL/min; the column temperature was 40°C; and the injection volume was 4 μL.

The information of these samples was qualitatively analyzed using the MetWare database, which was self-constructed by Metware Biotechnology Ltd (Jiaxing, China). Variable importance of predicted (VIP) ≥1.0 and absolute fold change (FC) ≥5.0 were used as criteria for differential metabolite selection. The identified metabolites were annotated using the KEGG compound database (http://www.kegg.jp/kegg/compound/), and then the annotated metabolites were mapped to the KEGG pathway database (http://www.kegg.jp/kegg/pathway.html).

### Data analysis

The effect of additives on silage quality and microbial diversity indices were analyzed using SPSS 20.0 (IBM Co., Armonk, NY, USA). Tukey’s multiple comparison test was used to assess the differences between the means. Silage performance data (DM, WSC, CP, NDF, ADF, pH, LA, AA, BA, and NH_3_-N parameters) microbial diversity indices (Shannon, Simpson, Ace, Chao1, and Coverage) were expressed as the mean ± standard error of three measurements.

## Results and discussion

### Characteristics of fresh materials before ensiling

**Figure 1:**
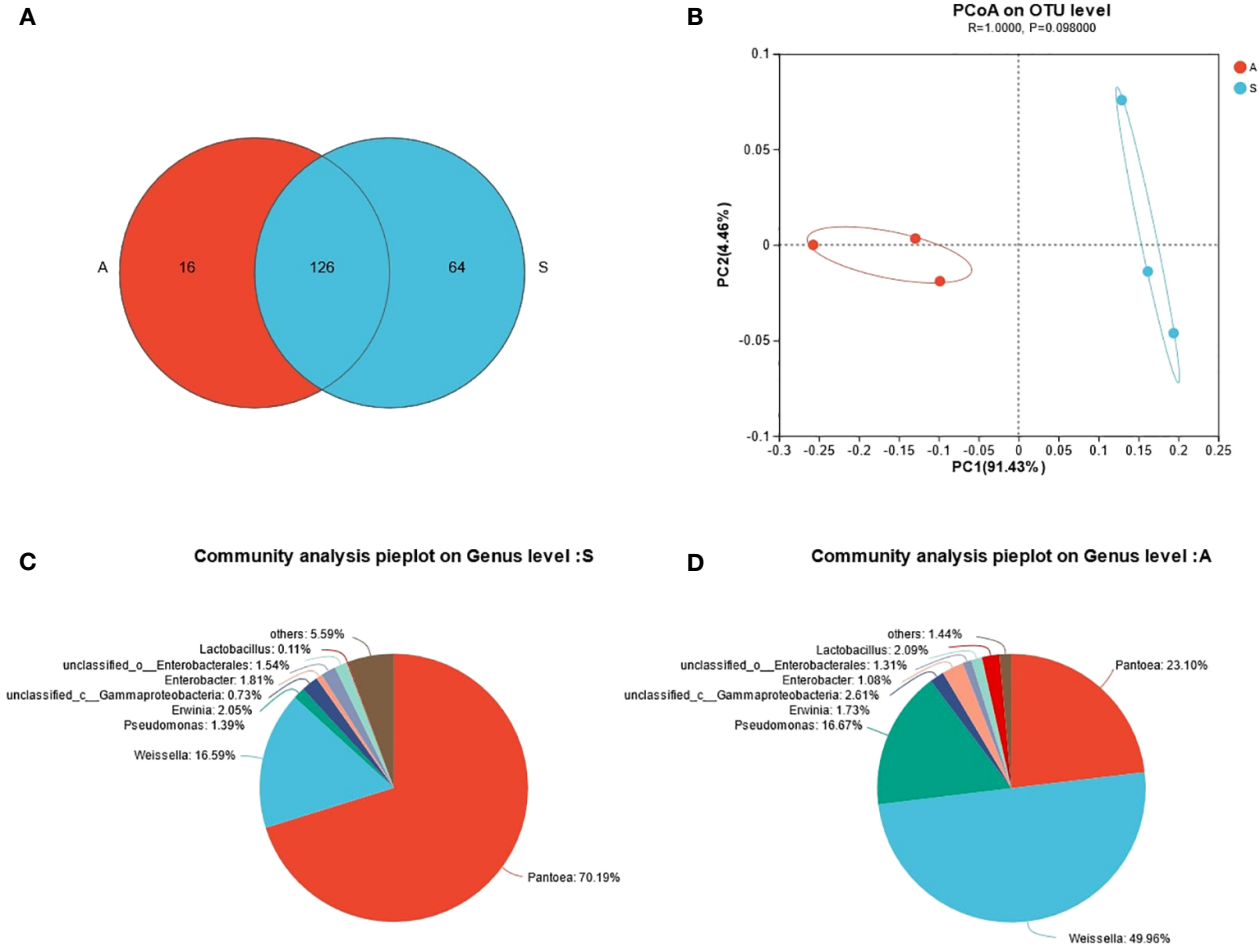
**(A)** Venn diagram of microbial diversity. **(B)** Principal coordinate analysis (PCoA) of bacterial community of fresh alfalfa and sunflower straw. **(C)** Microbial community composition of fresh sunflower straw at the genus level. **(D)** Microbial community composition of raw alfalfa at the genus level. A, alfalfa. S, sunflower straw.

As is shown in **Table 1**, the DM, CP, EE, NDF, ADF, WSC and ASH contents of sunflower straw and alfalfa were 53.95 and 56.30, 10.50 and 20.00, 3.43 and 2.77, 44.78 and 35.61, 38.90 and 28.70, 4.50 and 5.80, 10.14 and 10.68, respectively. The CP content of alfalfa was almost twice as much as that of sunflower straw. Furthermore, the NDF and ADF of alfalfa were lower than that of sunflower straw. However, the EE content of sunflower straw was higher. WSC played a vital role in the overall fermentation ecosystem, which also provided the necessary substrate for lactic acid production. The WSC content is the limiting factor for fermentation, and Zhang et al. (2010) concluded that in order to preserve fresh feedstuffs successfully, it is necessary the minimum WSC content about 30 g/kg of DM. In the present experiments, the fermentation conditions were met for both feedstuffs, notwithstanding the soluble carbohydrate content of both feedstuffs was low. It can also be observed that sunflower stalks carry a higher number of lactic acid bacteria compared to alfalfa, and have fewer coliforms and yeast counts. From **Figure 1A**, it can be seen that alfalfa contains 142 OTUs and sunflower straw contains 190 OTUs, of which the total number of OTUs is 126. From the **Figure 1B** we know that the two different feedstocks were separated to a greater extent, indicating that there was a greater difference in microbial diversity between them. As can be seen from the **Figure 1C**, the dominant flora of sunflower straw was *Pantoea* spp., which accounted for 70.19%, followed by *Weissella* spp., which accounted for 16.59%, and the proportion of *Lactobacillus* spp. only 0.11%. In contrast, the main dominant bacterial group of alfalfa was *Weissella* spp., accounting for 49.96%, followed by *Pantoea* spp. and *Pseudomonas* spp., accounting for 23.10% and 16.67%, respectively, and *Lactobacillus* spp. only accounted for 2.09%, **Figure 1D**. Notwithstanding alfalfa carried a low number of microorganisms, it had a high relative abundance of *Weissella* spp. and *Lactobacillus* spp. *Pseudomonas* spp. is one of the common soil bacteria that can survive under anaerobic conditions. The high abundance of *Pseudomonas* spp. in the alfalfa material might be due to soil contamination of the material.

**Table 1:** Microbial and chemical compositions of alfalfa and sunflower straw before ensiling.

	Sunflower straw	Alfalfa
DM,%FM	53.95 ± 2.17	56.30 ± 5.42
CP,%DM	10.50 ± 1.04	20.00 ± 0.66
EE,%DM	3.43 ± 0.55	2.77 ± 0.15
NDF,%DM	44.78 ± 2.01	35.61 ± 1.02
ADF,%DM	38.90 ± 1.78	28.70 ± 0.92
WSC,%DM	4.50 ± 0.36	5.80 ± 0.70
Ash,%DM	10.14 ± 0.63	10.68 ± 0.22
Lactic acid bacteria, log_10_ cfu/g FM	6.40 ± 0.10	6.04 ± 0.09
Coliform bacteria, log_10_ cfu/g FM	5.82 ± 0.05	6.05 ± 0.06
Yeasts, log_10_ cfu/g FM	4.13 ± 0.07	4.77 ± 0.19

DM, Dry matter; FM, Fresh matter; CP, Crude protein; EE, Ether Extract; NDF, Neutral detergent fiber; ADF, Acid detergent fiber; WSC, Water soluble carbohydrate; LAB, Lactic acid bacteria; cfu, colony-forming units. The same below.

(Mean ± SEM, N = 3).

### Analysis of the fermentation quality of mixed silage

**Figure 2:**
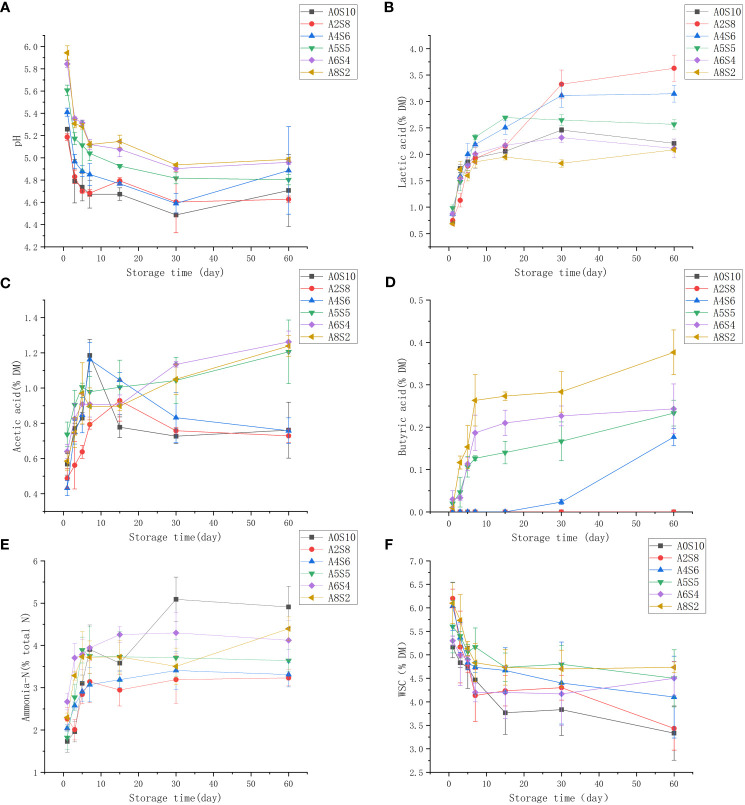
The dynamics of pH **(A)** and lactic acid **(B)** and acetic acid **(C)** and butyric acid **(D)** and ammonia-N **(E)** and WSC **(F)**.

The reason why the silage can be preserved for a long time is mainly based on the anaerobic as well as acidic environment; the decrease in pH is mainly caused by the metabolism of LAB, which can convert the water soluble carbohydrates to LA, while LA fermentation produces a high osmotic pressure in the environment, which deactivates microorganisms and then maintains the nutritive value of the fresh crop (Da Silva et al., 2017). The LA (pKa of 3.86) produced by LAB is usually the highest concentration acid in silage, which strength is 10 to 12 times higher than AA (pKa 4.75) and PA (pka 4.87). Therefore, LA contributes in decreasing pH levels during the fermentation pathways. (Kung et al., 2018). In addition, lactic acid-producing fermentations result in the lowest crop DM and energy losses during storage. The resistance of silage to pH reduction is called buffering capacity. This is exerted by compounds present in the crop such as crude proteins, inorganic ions, organic acids and other substances, greater buffering capacity requires greater WSC content and longer time for effective fermentation by lowering pH and inhibiting undesirable fermentation (Buxton et al., 2003). In addition, the most important substrate for fermentation is WSC. Also, some enzymes hydrolyze starch and hemicellulose to provide more hexoses and pentoses for microbial growth. Hexose monosaccharides, oligosaccharides and polysaccharides, such as glucose, fructose, sucrose and fructans, are the main WSC that are easily fermented (Da Silva et al., 2017). From this experiment, it was observed that the Different proportions of straw and alfalfa, the number of days of fermentation had a significant effect on pH, LA and AA content *(P<0.05)*. As a whole, the pH among the treatment groups decreased rapidly in the pre-fermentation period, then leveled off and increased slightly in the late fermentation period. The pH of the treatment groups with less alfalfa (A0S10、A2S8、A4S6) decreased more rapidly, reaching 4.67 on the 5th day of fermentation in A0S10, while the pH of the treatment groups with a higher proportion of alfalfa (A6S4、A8S2) decreased more slowly (**Figure 2A**). In general, legumes such as alfalfa possess higher buffer energy values and thus take longer to ferment. The rate of pH decline is considered to be a more important indicator of the kinetics in silage fermentation than the final pH (Mazza Rodrigues et al., 2008; Mu et al., 2020). Kennang et al. (2022) concluded that the rapid decline in pH during the early stages of fermentation is a key determinant of silage quality due to the inhibition of spoilage microorganisms from degrading proteins and producing NH_3_-N. The pH of A0S10、A2S8, and A4S6 decreased during the subsequent fermentation but the difference was not significant *(P>0.05)*, whereas A6S4 and A8S2 continued to decrease to some extent after 15 days of fermentation and the difference was significant *(P<0.05)* (**Figure 2A**).

LA accumulation (**Figure 2B**) was more rapid in the early stage, followed by a gradual slowdown of lactic acid accumulation. It is noteworthy that A2S8 and A4S6 still showed a substantial increase in lactic acid accumulation in the later stages of fermentation and had significantly higher lactic acid content than the rest of the treatments at the completion of 60 d of fermentation *(P<0.05)*. This might be due to the higher number of lactic acid bacteria carried on the feedstock of sunflower straw. Demirel et al. (2006) concluded that the organic acid content of sunflower silage was higher than sorghum silage compared to other mixed silages, and that the lactic acid content increased with the increase in the proportion of sunflower in sunflower-maize silage (Demirel et al., 2008), which is similar to the results of the present experiment.

Heterofermentation is dominated by heterofermentative lactic acid bacteria, Enterobacteriaceae, and Clostridia, which ferment soluble carbohydrates to acetic acid ultimately, which can result in some dry matter loss (Blajman et al., 2018). In this experiment, it was observed that the AA content in A5S5, A6S4, and A8S2 increased with the proportion of alfalfa (**Figure 2C**), while the acetic acid content in A0S10, A2S8, and A4S6 showed a downward trend in the latter stages of fermentation. Wang et al. (2018) concluded that the AA concentration gradually increased with increasing proportion of alfalfa, independent of the straw material. This may be attributed to the complex microbial community on alfalfa. Acetic acid bacteria may attach to alfalfa, which can metabolize fructose and glucose via the pentose phosphate pathway, with acetaldehyde acting as an intermediate to produce acetic acid before reaching anaerobic conditions (Wang et al., 2018). Butyric acid is undesirable in silage because it reduces intake by livestock if it exceeds 5 g/kg DM (Wang et al., 2019a). The presence of butyric acid indicates the metabolic activity of Clostridia. Butyric acid (**Figure 2D**) was not detected in A0S10 and A2S8 in the results of this experiment, and A4S6 detected butyric acid only on the 30th day. Meanwhile, the butyric acid content gradually increased as the proportion of alfalfa increased. This might cause faster decrease in silage pH, this might because the addition of sunflower straw inhibited the activity of Clostridium.

High concentrations of NH_3_-N in silage are a sign of excessive protein breakdown, usually caused by a slow decrease in pH (Kung and Shaver, 2001). The cause is a combination of plant proteases and microorganisms. Both Clostridium and plant protein hydrolases are active at pH 5.0 to 6.0 (Wang et al., 2019a). NH_3_-N concentrations are positively correlated with the relative abundance of Enterobacteriaceae spp. Du et al. (2022) suggest that Enterobacteriaceae compete for nutrients and produce NH_3_-N.The relatively high NH_3_-N of A6S4 and A8S2 in this trial may be due to the slow decline in pH (**Figure 2E**). Soluble carbohydrates, serving as substrates for lactic acid bacteria fermentation, were continuously decreasing throughout the fermentation process (**Figure 2F**).

### Chemical compositions of mixed silages

The feed value of ensiled feedstuff is influenced by various factors, such as Neutral Detergent Fibre, carbohydrates, fats, and protein content of the feed (Bal et al., 1997). As can be seen from **Table 2**, with the increase of alfalfa ratio in all treatments, the CP gradually increases, an outcome that can be anticipated. Research conducted by Gholami-Yangije et al. (2019) suggests that ensiling can increase the crude protein content in sunflower residues. This aligns with the results of our experiment, where there is a gradual increase in crude protein content during the fermentation of A2S8 and A4S6 *(p>0.05).* This increment might be due to the synthesis of microbial body protein during the fermentation process. Conversely, in A8S2 and A6S4, the crude protein content gradually decreases *(p>0.05)*. Studies indicate that, during the ensiling process, protein hydrolysis typically begins with natural plant protein enzymes. These enzymes break down proteins into peptides and free amino acids, which are then further degraded to amides, amines, and ammonia through microbial activity (Kung et al., 2018). Li et al. (2018) propose that different feedstuff will result in varying protease activities in the ensiling process, such as carboxypeptidase, aminopeptidase and acid protease, which in turn leads to different levels of protein hydrolysis. Therefore, in this experiment, the varying patterns in CP changes among different treatments may be due to differences in feedstuff ratios, which cause varied protease activities. Simultaneously, the role of microbes, such as Clostridium perfringens and Escherichia coli, also exert certain impacts. The complex structure of lignocellulose has consistently been identified as a limitation factor for the feed conversion of sunflower straws. This part of the material is often difficult for microbes to utilize directly during the fermentation process. Research indicates that ensiling sunflower stalks can decrease the content of NDF and increase Acid Detergent Lignin (ADL) (Gholami-Yangije et al., 2019). These findings are inconsistent with our experiment. In our experiment, during the fermentation process, the NDF content in A5S5, A6S4 and A8S2 gradually reduced, while the NDF content in the remaining groups remained basically unchanged or slightly increased. Simultaneously, the ADF content declined to varying degrees across all groups, with the most noticeable drop observed in A0S10 and A5S5 groups. This could possibly be due to the degradation of cellulose induced by the additives.

**Table 2:** Dynamic changes in the chemical composition of mixed silage. (Mean ± SEM, N = 3).

		1d	3d	5d	7d	15d	30d	60d	p	SEM	D	T	D*T
DM	A0S10	32.83 ± 0.42fA	33.08 ± 1.12bA	33.73 ± 0.52dA	33.26 ± 1.00dA	33.32 ± 0.20dA	33.62 ± 0.36cA	33.25 ± 1.11dA	0.65	0.1594	0.006	<0.0001	0.633
	A2S8	34.3 ± 0.91eAB	33.37 ± 0.70bB	34.40 ± 0.37cdAB	34.18 ± 0.36cdAB	33.62 ± 0.63dAB	34.56 ± 0.21cA	33.69 ± 0.55dAB	0.16	0.14			
	A4S6	39.38 ± 0.27aA	37.70 ± 0.65aA	38.55 ± 0.85aA	38.55 ± 2.22aA	38.13 ± 0.47aA	38.30 ± 1.22aA	37.56 ± 1.01aA	0.54	0.24			
	A5S5	35.36 ± 0.76dA	36.31 ± 2.19aA	35.02 ± 0.70cA	35.60 ± 0.65bcA	35.04 ± 0.85cA	35.79 ± 0.23bA	34.99 ± 0.38cA	0.65	0.21			
	A6S4	36.65 ± 0.53cAB	36.69 ± 0.26aAB	36.54 ± 0.62bB	36.79 ± 0.51abAB	36.66 ± 0.88bAB	37.62 ± 0.25aA	36.07 ± 0.24bcB	0.09	0.13			
	A8S2	38.23 ± 0.35bA	37.63 ± 0.30aAB	37.41 ± 0.48bBC	36.92 ± 0.29abBC	36.85 ± 0.16bBC	38.27 ± 0.71aA	36.75 ± 0.40abC	0.001	0.15			
	p	<0.0001	<0.0001	<0.0001	0.001	<0.0001	<0.0001	<0.0001					
	SEM	0.555	0.5052	0.4308	0.4791	0.4422	0.4434	0.4039					
CP	A0S10	13.33 ± 0.12fA	12.6 ± 1.60dA	12.5 ± 0.87fA	12.77 ± 1.01eA	11.63 ± 0.42eA	12.77 ± 0.12fA	12.77 ± 1.10dA	0.49	0.1961	0.004	<0.0001	0.613
	A2S8	13.90 ± 0.20eB	14.27 ± 0.12cAB	14.10 ± 0.10eAB	14.07 ± 0.25dAB	14.37 ± 0.15dAB	14.63 ± 0.68eA	14.60 ± 0.17cA	0.075	0.0796			
	A4S6	16.00 ± 0.10dAB	15.47 ± 0.21cB	16.07 ± 0.29dAB	16.20 ± 0.30cAB	15.83 ± 0.31cAB	16.10 ± 0.62dAB	16.67 ± 1.33bA	0.399	0.1323			
	A5S5	17.47 ± 0.42cA	17.03 ± 0.47bAB	17.10 ± 0.36cAB	17.63 ± 0.61bA	16.57 ± 0.15cB	17.37 ± 0.35cA	17.60 ± 0.17bA	0.052	0.1064			
	A6S4	19.53 ± 0.23bA	18.63 ± 0.40aAB	18.77 ± 0.38bAB	19.13 ± 0.49aAB	18.47 ± 1.10bB	18.57 ± 0.35bAB	19.33 ± 0.25aAB	0.172	0.13			
	A8S2	20.30 ± 0.00aA	19.73 ± 0.42aA	20.00 ± 0.53aA	19.93 ± 0.21aA	20.07 ± 0.40aA	20.10 ± 0.30aA	19.97 ± 0.25aA	0.604	0.0718			
	p	<0.0001	<0.0001	<0.0001	<0.0001	<0.0001	<0.0001	<0.0001					
	SEM	0.6372	0.6136	0.6309	0.6347	0.6693	0.5967	0.6287					
NDF	A0S10	44.70 ± 1.02aA	44.47 ± 3.87aA	44.82 ± 2.51aA	43.6 ± 1.76aA	45.31 ± 0.85aA	44.40 ± 0.53aA	46.71 ± 3.77aA	0.809	0.4833	0.458	<0.0001	0.2
	A2S8	42.67 ± 0.73bAB	41.59 ± 0.61abB	43.28 ± 1.13abAB	42.77 ± 0.37aAB	41.66 ± 0.81bB	43.90 ± 1.90aA	41.77 ± 1.03bcB	0.108	0.2648			
	A4S6	41.81 ± 0.47bcAB	41.98 ± 0.46abAB	41.15 ± 0.95bcdAB	40.26 ± 0.42bB	41.58 ± 0.64bAB	41.19 ± 1.66bAB	42.58 ± 1.70bA	0.245	0.2434			
	A5S5	40.10 ± 0.35dA	39.70 ± 0.28bA	39.67 ± 0.54dA	40.24 ± 1.03bA	40.03 ± 0.49cdA	39.22 ± 0.49bcAB	38.46 ± 0.51cB	0.024	0.1653			
	A6S4	40.97 ± 0.44cdBC	41.81 ± 0.56abAB	42.42 ± 0.46abcA	40.53 ± 0.10bC	41.26 ± 1.28bcBC	41.10 ± 0.30bBC	40.62 ± 0.35bcC	0.021	0.1775			
	A8S2	39.88 ± 0.17dABC	40.35 ± 0.69bAB	40.62 ± 1.44cdA	39.09 ± 0.63bBC	38.69 ± 0.57dC	38.77 ± 0.94cC	38.89 ± 0.49cBC	0.04	0.2203			
	p	<0.0001	0.056	0.006	<0.0001	<0.0001	<0.0001	0.001					
	SEM	0.4178	0.4905	0.4969	0.421	0.5176	0.5626	0.7525					
ADF	A0S10	39.73 ± 0.45aA	37.93 ± 1.95aAB	37.43 ± 0.29aAB	36.67 ± 0.59aB	36.2 ± 1.59abB	35.67 ± 0.40abB	35.73 ± 1.99abcB	0.015	0.3781	<0.0001	<0.0001	0.001
	A2S8	37.77 ± 0.93bA	36.63 ± 0.84abAB	36.53 ± 1.64aAB	35.70 ± 1.31aAB	34.57 ± 0.50bcB	36.13 ± 1.56abAB	36.60 ± 1.71abAB	0.182	0.3108			
	A4S6	36.83 ± 0.55bA	36.13 ± 1.55abA	36.60 ± 0.36aA	36.83 ± 0.67aA	35.77 ± 0.40abcA	36.60 ± 1.49aA	37.00 ± 0.53aA	0.67	0.1914			
	A5S5	39.17 ± 0.21aA	37.80 ± 0.78aAB	36.40 ± 1.44abBC	36.13 ± 0.40aBC	37.30 ± 1.51aABC	35.50 ± 1.71abC	33.20 ± 0.69dD	0	0.4407			
	A6S4	35.53 ± 0.96cB	37.43 ± 0.81aA	35.97 ± 0.86abB	35.70 ± 0.70aB	35.03 ± 0.91bcB	35.20 ± 0.44abB	34.67 ± 0.40bcdB	0.012	0.2315			
	A8S2	34.37 ± 0.61cA	34.70 ± 0.30bA	34.50 ± 0.87bA	33.63 ± 0.23bA	33.87 ± 0.90cA	34.17 ± 0.74bA	33.67 ± 0.23cdA	0.293	0.1425			
	p	<0.0001	0.042	0.078	0.002	0.024	0.266	0.007					
	SEM	0.4784	0.3581	0.2987	0.2933	0.3447	0.2995	0.4122					
EE	A0S10	2.67 ± 0.35bcA	2.00 ± 0.85cA	2.07 ± 0.21bA	2.07 ± 0.25cA	1.87 ± 0.64dA	2.20 ± 0.26eA	1.8 ± 0.61cA	0.494	0.1099	<0.0001	<0.0001	<0.0001
	A2S8	2.17 ± 0.25dB	2.00 ± 0.70cB	2.40 ± 0.10bAB	2.27 ± 0.12cAB	2.17 ± 0.31cdB	2.56 ± 0.32deAB	2.83 ± 0.06bA	0.11	0.0844			
	A4S6	2.40 ± 0.20cdA	2.43 ± 0.32bcA	2.43 ± 0.25bA	2.33 ± 0.38cA	2.40 ± 0.36cdA	3.03 ± 0.35cdA	2.87 ± 0.75bA	0.297	0.0938			
	A5S5	3.17 ± 0.31aBC	3.53 ± 0.12aB	2.50 ± 0.52bD	3.07 ± 0.15bBCD	2.80 ± 0.26bcCD	3.40 ± 0.53bcBC	4.20 ± 0.30aA	0.001	0.1301			
	A6S4	3.07 ± 0.21abB	3.10 ± 0.20abB	3.10 ± 0.30aB	3.77 ± 0.25aA	3.57 ± 0.51abAB	3.80 ± 0.10bA	4.17 ± 0.45aA	0.004	0.1067			
	A8S2	2.90 ± 0.10abD	3.13 ± 0.51abD	3.37 ± 0.38aCD	3.80 ± 0.00aBC	4.07 ± 0.59aAB	4.60 ± 0.30aA	4.23 ± 0.15aAB	0	0.1436			
	p	0.002	0.014	0.003	<0.0001	0.001	<0.0001	<0.0001					
	SEM	0.0996	0.1768	0.125	0.1774	0.2099	0.2032	0.2407					

Different uppercase letters within the same column in the table indicate significant differences among treatments at the same fermentation time (P < 0.05); different lowercase letters in the table indicate significant differences over different times within the same treatment (P < 0.05).

### Microbial abundance and biodiversity in mixed silage

**Figure 3:**
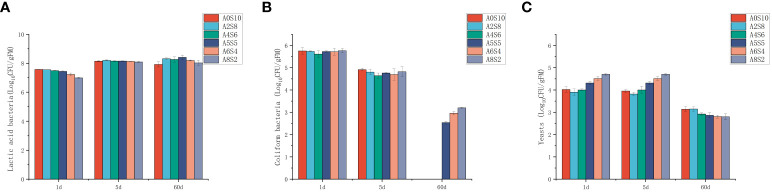
Quantification of Lactic acid bacteria **(A)**, Coliform bacteria **(B)**, and Yeasts **(C)** populations in mixed silage on1d,5d and 60d.

**Figure 4:**
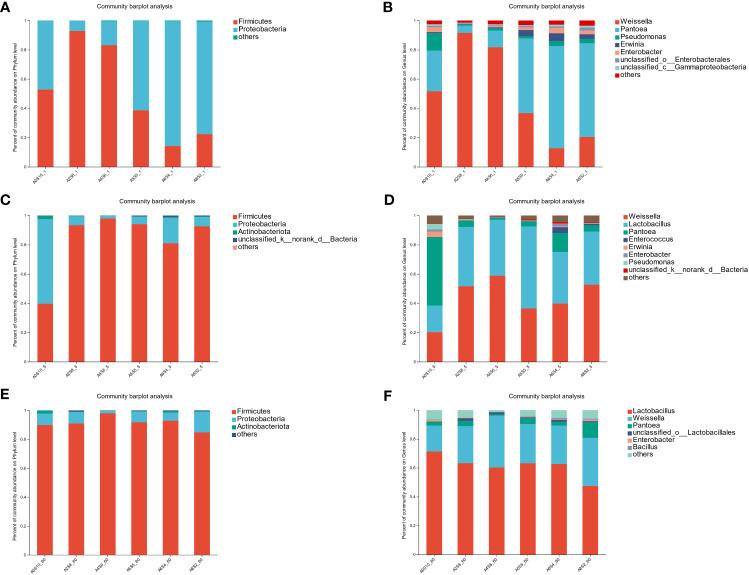
Phylum-level composition of bacterial communities in mixed silage on 1d **(A)**, 5d **(C)**, and 60d **(E)**. Genus -level composition of bacterial communities in mixed silage on 1d **(B)**, 5d **(D)**, and 60d **(F)**.

The fermentation process of silage feed is quite complex, involving a variety of microbial types, and ultimately leading to different fermentation outcomes. The community structure, species diversity, and functions of the microbes are critical factors impacting silage fermentation (Besharati et al., 2020). Among them, LAB ferment soluble carbohydrates to produce LA and other beneficial organic acids, subsequently reducing pH to inhibit the growth of harmful microbes. Consequently, during the silage fermentation process, the succession of microbes typically revolves around the interactions between LAB and harmful microbial entities (Besharati et al., 2022). As seen in **Figure 3**, during the fermentation process, the number of LAB significantly increased, while the numbers of Coliform bacteria and Yeasts considerably decreased in all groups. After 60 days of fermentation, the quantity of Coliform bacteria systematically decreased with the increment in the proportion of sunflower straws, particularly in the groups A0S10 and A2S8. The Coliform bacteria count in these groups were lower than 100 cfu/g. This demonstrates that the addition of sunflower straws effectively reduces the Coliform bacteria count. In China, both the pith and leaves of sunflower straw can be used for medicinal applications. Modern research indicates that sunflower straw and leaves contain numerous bioactive compounds, such as terpenoids, lignans, and flavonoids (Amakura et al., 2013; Torres et al., 2015). These may account for their effective inhibition of Coliform bacteria. LAB belong to the Firmicutes phylum, which includes several genera such as *Lactobacillus* spp., *Enterococcus* spp., and *Lactococcus* spp. (Besharati et al., 2021b). In the phylum level analysis of the experiment (**Figure 4**), all treatment groups showed an increase in Firmicutes as fermentation progressed. The shift from Proteobacteria to Firmicutes is a normal occurrence during the ensiling process, as the environment shifts from aerobic to anaerobic. The anaerobic and low pH conditions favor the growth of Firmicutes. Bacteria within the Firmicutes produce acid in an anaerobic environment and can secrete a variety of enzymes (Besharati et al., 2023). At the genus level, we observed that *Weissella* spp. dominated initially in all treatment groups, followed by *Lactobacillus* spp. As fermentation progressed, the relative abundance of *Lactobacillus* spp. gradually surpassed *Weissella* spp. Previous reports suggest that *Weissella* spp., *Pediococcus* spp., and *Lactococcus* spp. initiate the ensiling fermentation but then become less vigorous. As fermentation time lengthens, the primary bacteria gradually shift toward those that are more tolerant of low pH, such as *Lactobacillus* spp. In our trial, the fermentation process was mainly initiated by *Weissella* spp., with no observations of *Pediococcus* spp. and *Lactococcus* spp. On the first day of fermentation, the highest relative abundance of *Weissella* spp. was observed in the A2S8 and A4S6 groups (91.58% and 81.74% respectively), while the dominant bacteria in the A5S5, A6S4, and A8S2 groups were *Pantoea* spp. (50.88%, 69.75%, and 63.96% respectively), followed by *Weissella* spp. (36.80%, 12.78%, and 20.49% respectively) (**Figure 4**). This evidences that the addition of a higher proportion of sunflower straw can initiate mixed ensiling fermentation earlier.

*Pantoea* spp. is the most common facultative aerobic genus in fresh materials. In our experiment, *Pantoea* spp. decreased rapidly, with A2S8, A4S6, A5S5, A6S4, and A8S2 decreasing to 4.45%, 1.15%, 3.52%, 12.92%, and 4.39% respectively by day 5 of fermentation. This is due to the high sensitivity of *Pantoea* spp. to pH decline. The current role of *Pantoea* spp. species in ensiling fermentation is not yet clear. However, according to previous research, *Pantoea* spp. may reduce NH_3_-N. Because the fermentation of A5S5, A6S4, and A8S2 started later and alfalfa material contained more miscellaneous bacteria (*Pseudomonas* spp.*, Ewinia* spp.*, Enterobacter* spp.), a considerable number of *Pseudomonas* spp., *Erwinia* spp., and *Enterobacter* spp. were present on both the first and fifth days. *Pseudomonas* spp. is believed to consume protein in silage. *Erwinia* spp. is considered the major bacterium causing spoilage in fresh plants, where in silage, *Erwinia* spp. competes with LAB for fermentation substrate (McGarvey et al., 2013). *Enterobacter* spp. can ferment glucose and LA to form AA and ethanol, and degrade proteins to ammonia (Borreani et al., 2018). Therefore, these bacteria should be inhibited in silage. During subsequent fermentation, *Weissella* spp. and *Lactobacillus* spp. gradually acquire a position of dominance. However, groups A6S4 and A8S2 still contain a relatively high abundance of *Pantoea* spp. and *Enterobacter* spp. This might be due to the fact that in these two treatments, the pH decreased slowly, which prevented the quick suppression of harmful bacteria. We also noted that throughout the entire fermentation process, the relative abundance of *Lactobacillus* spp. in group A0S10 was persistently low. This may be owing to the fact that LAB usually require a variety of amino acids and vitamins for growth. Perhaps the protein content in sunflower straw was too low to promote their reproduction and growth (Besharati et al., 2021a).

### Metabolites of mixed silage

Microorganisms influence the quality of silage feed through the production of a series of metabolites. These metabolites play multiple roles in silage feed, such as improving fermentation quality and flavor, as well as extending aerobic stability (Hu et al., 2020). Hu et al. (2020) detected a total of 196 metabolites in alfalfa silage, primarily composed of organic acids, polyhydric alcohols, ketones, and aldehydes. To our knowledge, reports on the metabolites present in sunflower stems and leaves are considerably limited. In our experiment, the metabolomics analysis identified 2313 compounds in sunflower and alfalfa raw materials, solely sunflower silage, and a mixture of sunflower and alfalfa silage. These were comprised of 176 types of amino acids and their derivatives, 292 phenolic acids, 77 nucleotides acids and their derivatives, 445 flavonoids, 34 quinones, 105 lignans and coumarins, 13 tannins, 208 alkaloids, 330 terpenoids, 136 organic acids, 197 lipids (**Supplementary Table S1**). In our research, flavonoids were the most abundant, found in considerable quantities in both types of raw materials and in the silage.

The differences in metabolites between alfalfa and sunflower stalks, as illustrated in **Supplementary Table S2**, primarily lie in terpenoids, flavonoids, amino acid and derivatives, as well as lignans and coumarins. It is generally believed that alfalfa is rich in flavonoids, mainly tricin and apigenin. Alfalfa flavonoids are a mixture of forms acylated with hydroxycinnamic acid derivatives (ferulic, coumaric, and sinapic acid), and nonacylated (Rafińska et al., 2017). The principal biological function of flavonoids is their antioxidant action. Research by Chiurazzi et al. (2022) indicates that alfalfa flavonoids can effectively enhance the lipid and oxidative metabolism in broilers. Lui et al. (2020) propose that the flavonoids in alfalfa mainly include apigenin, digloflavone, kainic acid, quercetin, and myricetin. Apigenin possesses anticancer, antioxidant, and anti-inflammatory effects. Currently, studies on the antibacterial effect of apigenin are mainly conducted under aerobic conditions, with its antibacterial function being quite limited under anaerobic conditions (Wang et al., 2019b). Present research on sunflower stalks using metabolomics is rather limited, but this study has identified substances such as nevadensin, penduletin, farrerol, rivularin*, and tenaxin in sunflower stalks. From the raw material perspective, compared to fresh alfalfa, fresh sunflower stalks have 9 kinds of sesquiterpenes down-regulated and 27 kinds of sesquiterpenes up-regulated. Among these up-regulated metabolites are nootkatone, artemisiifolin, pechueloic acid, and atractylenolide III. Nootkatone is considered one of the primary compounds responsible for the scent and taste of grapefruit. It is a high-value aromatic compound, also known for its insect repellent and anti-inflammatory properties (Li et al., 2021). Accordingly, the addition of sunflower stalks might possibly improve the flavor of silage. Atractylenolide III has pharmacological properties, including blood sugar and fat regulation, anti-thrombocyte, anti-osteoporosis, and antibacterial activities. It is especially renowned for its significant anti-inflammatory and neuroprotective effects (Deng et al., 2021). Meanwhile, compared to fresh alfalfa, fresh sunflower stalks have 10 types of lignans up-regulated and 3 types of lignans down-regulated. Lignans are mainly found in the woody parts of plants, possessing a variety of biological activities including antioxidant, antibacterial, and antiviral effects. Overall, the metabolite content in alfalfa is higher than in sunflower stalks. The relatively high metabolites in sunflower stalks are primarily flavonoids and sesquiterpenes, where their significant effects are concentrated on medicinal activities like anti-inflammatory and anticancer properties.

**Figure 5:**
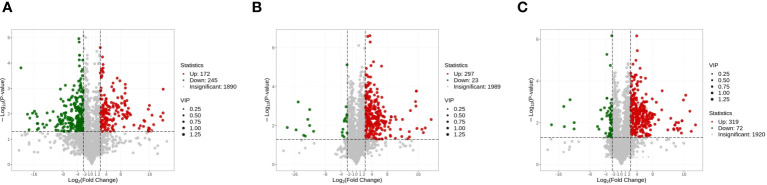
**(A)** Volcano plot analysis of alfalfa before ensiling VS sunflower straw; **(B)** Volcano plot analysis of A0S10 VS A5S5; **(C)** Volcano plot analysis of A0S10 VS A6S4.

Interestingly, after 60 days of fermentation, most of the increased metabolites in the sunflower raw materials have disappeared. It was observed that compared to A0S10, A5S5 had 298 metabolites up-regulated and 23 metabolites down-regulated (**Supplementary Table S3**). In comparison to A0S10, A6S4 had 320 metabolites up-regulated and 72 metabolites down-regulated (**Supplementary Table S4**; **Figure 5**). Noteworthy is that the higher parts of flavonoids and sesquiterpenes observed in the sunflower stalks in raw materials were not detected after fermentation. All types of metabolites in the two mixed storages (amino acids and their derivatives, phenolic acids, nucleosides and their derivatives, flavonoids, quinones, lignans and coumarins, tannins, alkaloids, terpenes, and organic acids) were higher than in the pure sunflower storage. The main part of these were flavonoids, including tricetin, tricin, pseudobaptigenin, and vestitol. Tricetin and vestitol have anti-inflammatory properties (Geraets et al., 2007; Franchin et al., 2016). Tricin has pharmacological activities of anti-inflammation, antioxidant, and antiviral (Lam et al., 2021). It’s worth noting that there were no quinones in the differential metabolites of the two raw materials before silage, mainly anthraquinones. However, compared to the pure sunflower storage, there were 15 and 7 types of anthraquinones up-regulated in the two mixed storages, respectively. Anthraquinones possess pharmacological and toxicological effects, primarily including anti-hyperlipidemia, anti-cancer, immune regulation, and purgative effects (Wang et al., 2021a). Among them, emodin possesses anti-cancer and anti-inflammatory effects, and inhibits Gram-positive bacteria, especially *Bacillus subtilis* and *Staphylococcus aureus*. However, excessive consumption of emodin can lead to hepatotoxicity, nephrotoxicity, and reproductive toxicity (Dong et al., 2016). Additionally, significant up-regulation in metabolites also included laccaic acid D, isoemodin, and aloe emodin. Simultaneously, two treatment groups each had 8 and 27 kinds of amino acids and their derivatives up-regulated respectively.

**Figure 6:**
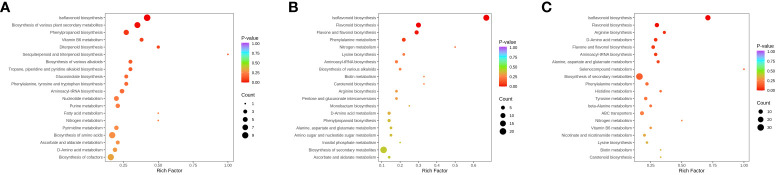
KEGG pathway enrichment analysis of differentially accumulated metabolites. **(A)** Alfalfa before ensiling VS sunflower straw; **(B)** A0S10 VS A5S5; **(C)** A0S10 VS A6S4.

Enrichment analysis of Kyoto Encyclopedia of Genes and Genomes (KEGG) pathways (**Figure 6**) indicates that the differences in sunflower stalks and alfalfa raw materials mainly concentrate on Isoflavonoid biosynthesis, Biosynthesis of various plant secondary metabolites, Phenylpropanoid biosynthesis, Biosynthesis of amino acids, and Biosynthesis of cofactors. Differential metabolites between A0S10 and A5S5 primarily focus on Isoflavonoid biosynthesis and Flavonoid biosynthesis. Differential metabolites between A0S10 and A6S4 mainly focus on Isoflavonoid biosynthesis and Biosynthesis of secondary metabolites.

## Conclusion

When sunflower straw was ensiled alone, the results showed compromised fermentation quality and microbial diversity. However, with a higher proportion of sunflower straw added, the co-ensiling process effectively accelerated the rate of pH reduction, minimized nutrient loss during the early phase of fermentation, and lowered the concentrations of acetic and butyric acids. In addition, it rapidly decreased the relative abundance of miscellaneous bacteria and enhanced the relative abundance of lactic acid bacteria during the early stages of fermentation. Furthermore, compared to ensiling sunflower straw alone, co-ensiling resulted in the upregulation of several metabolites.

For requirements for a specific article type please refer to the Article Types on any Frontiers journal page. Please also refer to Author Guidelines for further information on how to organize your manuscript in the required sections or their equivalents for your field.

## Data availability statement

The original contributions presented in the study are included in the article/**Supplementary Material**. Further inquiries can be directed to the corresponding authors.

## Author contributions

HJ: Conceptualization, Formal analysis, Investigation, Methodology, Writing – original draft. S-YW: Investigation, Methodology, Writing – review & editing. H-RW: Methodology, Writing – review & editing. Y-YJ: Data curation, Investigation, Writing – review & editing. HQ: Data curation, Writing – review & editing. LS: Data curation, Writing – review & editing. JW: Formal analysis, Writing – review & editing. F-QG: Funding acquisition, Supervision, Writing – review & editing. BL: Funding acquisition, Supervision, Writing – review & editing.
